# Agitated Saline Contrast Echocardiography in the diagnosis of right to left shunts: Guidance and recommendations from the British Society of Echocardiography

**DOI:** 10.1186/s44156-026-00117-3

**Published:** 2026-06-01

**Authors:** Bushra Rana, Shaun Robinson, Liam Ring, Dave Oxborough, Mark Belham, Jim Newton, Alfa Ali, Nilesh Sutaria, Nina Bual, Martin Swaans, Nina Wunderlich, Victoria Delgado, David Northridge, Dave Hildick-Smith, Neil Ruparelia, Mark Turner, Michael Mullen, Chris Baker, Brian Clapp, Mark Spence, Horst Sievert, Iqbal Malik, Roxy Senior, Anjana Siva

**Affiliations:** 1 Guys and St Thomas’ Hospital NHS Foundation Trust, London, UK; 2 Imperial College Healthcare NHS Trust, London, UK; 3 West Suffolk Hospital NHS Foundation Trust, Bury St Edmunds, Suffolk, UK; 4 Research Institute for Sports and Exercise Science, Liverpool John Moores University, Liverpool, Merseyside, UK; 5 Addenbrookes Hospital, Cambridge University Hospitals NHS Foundation Trust, Cambridge, United Kingdom; 6 The John Radcliffe Hospital, Headley Way, Oxford, UK; 7 Department of Cardiology, St. Antonius Hospital, Nieuwegein, the Netherlands; 8 Department of Cardiology/Angiology, Asklepios Klinik Langen, Langen, Germany; 9 Hospital University Germans Trias i Pujol, Badalona, Spain; 10 Institute for Health Science Research Germans Trias i Pujol (IGTP), Badalona, Spain; 11 Edinburgh Royal Infirmary, Edinburgh, Scotland, UK; 12 Royal Sussex County Hospital, Brighton, UK; 13 University Hospital Bristol NHS Foundation Trust, Bristol, UK; 14 St Bartholomew’s Hospital, London, UK; 15 Mater Private Hospital, RCSI University of Medicine & Health Sciences, Dublin, Ireland; 16 CardioVascular Center Frankfurt, Frankfurt, Germany; 17 Royal Brompton Hospital, London, UK; 18 Queen Alexander Hospital, Portsmouth, UK

**Keywords:** Bubble contrast, Agitated saline, Transthoracic echocardiogram, Patent foramen ovale

## Abstract

Contrast echocardiography encompasses two forms of ultrasound contrast agents. This article focuses on agitated saline (AS-C) contrast, commonly called ‘bubble’ contrast. Its main application is in identifying the presence of a right to left shunt in a number of clinical syndromes, often associated with a patent foramen ovale (PFO). Dependant on the clinical presentation, the presence of a PFO with a likelihood of a causal role may be considered for percutaneous PFO closure. This guideline outlines a structured approach to performing an AS-C transthoracic echocardiogram, including the indications, protocols, and potential pitfalls for performing AS-C transthoracic echocardiography (TTE). It also addresses when to proceed to more advanced imaging modalities and highlights clinical decision-making frameworks such as the RoPE score.

**Supplementary Information:**

The online version contains supplementary material available at https://doi.org/10.1186/s44156-026-00117-3.

## Introduction

Contrast echocardiography encompasses two forms of ultrasound contrast agents. The first type refers to microbubbles containing inert bio-compatible high-molecular weight gas encapsulated within a shell, able to transverse the pulmonary capillary bed (PCB) to enable chamber visualisation through enhancing endocardial delineation and myocardial blood flow assessment. This subject is covered in the separate British Society of Echocardiography (BSE) guideline on echo contrast [1]. The second form of ultrasound contrast agent is agitated saline (AS-C) contrast commonly called ‘bubble’ contrast, where microbubbles are too large to cross the PCB. Its main application is in identifying the presence of a right to left shunt, in particular a patent foramen ovale (PFO). This article will describe the clinical application of AS-C in the diagnosis of right to left shunts, the indications, transthoracic echocardiography protocol and potential limitations.

AS-C is mainly used in the assessment of a right to left shunt (R to L shunt). The commonest presentation is ischaemic stroke, where the brain imaging typically displays a characteristic pattern suggestive of a cardioembolic source. In the absence of left-heart source (including AF, mitral stenosis, left ventricle (LV) thrombus, myxoma or vegetations), the possibility of a paradoxical embolism (where a venous thrombus enters the systemic arterial circulation through a right to left shunt) may need to be considered. Paradoxical embolism is suspected in the presence of a patent foramen ovale (or atrial septum defect) or a pulmonary arteriovenous malformation. The location of the shunt is categorised as intra or extra-cardiac respectively. Several other clinical syndromes are associated with R to L shunts most commonly due to a PFO and include decompression illness, arterial deoxygenation syndrome and migraine. In addition to the diagnosis of a R-L shunt, AS-C can be utilized for the diagnosis of a persistent left sided SVC, confirmation of entry into the pericardial space during pericardiocentesis and enhancement of the continuous wave Doppler signal of tricuspid regurgitation where it cannot be clearly seen.

### Role of transthoracic echocardiography in left circulation thromboembolism

Although transthoracic echocardiography (TTE) is not recommended routinely in patients following stroke, it is considered where findings are likely to change clinical management. TTE should be considered when:

- Clinical presentation and or clinical examination suggests underlying cardiac disease, including a new heart murmur or AF.
- Abnormal ECG (suggestive of underlying structural cardiac disease) not previously investigated.
- Other signs of systemic thromboembolism may be the initial presentation in patients with endocarditis. Typical concomitant findings include fever with a new cardiac murmur and systemic emboli e.g. wedge-shaped infarctions of organs such as the kidney or spleen.

Beyond the cardiovascular assessment, there are clinical and brain imaging characteristics which raise the suspicion of a cardiac source of embolism. These are summarised in Tables 1 and 2. 30% of ischaemic strokes result from a cardiac source. When the source of a suspected embolic stroke remains undetermined, the next step is to consider the possibility of paradoxical embolism (Table 3) and determine if a AS-C TTE is indicated.

**Table 1:** Brain imaging characteristics in cardioembolic strokeTable adapted from EACVI cardiovascular Imaging for the detection of embolic sources. Cohen A 2021

Brain imaging characteristics	Comment
Cortical infarcts	Usually considered embolic
Sub-cortical	May be linked, especially if large
Small sub-cortical infarcts (*lacunar*)	*Uncertain significance*
Infarcts involving multiple sites	Usually considered embolic
Anterior-posterior circulation Bilateral (both hemispheres) Multiple	Previous infarctions in multiple arterial distributions Without significant carotid or vertebrobasilar disease

**Table 2:** Suspicious clinical scenarios suggestive of a cardioembolic source of stroke

Clinical presentation characteristics
TIA symptoms without CV risk factors in a patient aged < 60years
Preceding Valsalva-like manoeuvrer immediately prior to stroke or TIA symptoms (e.g. straining on toilet or lifting a heavy weight such as a suitcase)
Presentation of a myocardial infarction with coronary artery thrombosis in the absence of underlying atherosclerotic disease
Simultaneous thromboembolism (e.g. deep vein thrombosis and or pulmonary embolism) around the same time as a left circulation thromboembolism. Suspect if recent period of immobilisation (e.g. surgery) or long travel.

**Table 3:** Criteria for stroke of undetermined source (cryptogenic)

Criteria for stroke of undetermined source (cryptogenic)
Brain imaging suggestive of distal source of embolism (*see* Table 2)
No evidence of a local source i.e. significant small or large cerebral vessel atherosclerosis
No evidence of other more rare causes e.g. carotid artery dissection, vasculitis, hypercoagulable state
No evidence of left heart source of embolism e.g. AF, LV thrombus, mitral stenosis, endocarditis

### Role of agitated saline contrast transthoracic echocardiography

The role of AS-C TTE is in determining the presence of an intra or extra cardiac shunt leading to a paradoxical embolism or less commonly hypoxaemia syndromes. Table 4 summarises the indications and includes uses additional to the diagnosis of R to L shunts. Table 5, summarises the causes of arterial deoxygenation syndrome, where R to L shunting allows deoxygenated blood to enter the left arterial circulation resulting in hypoxaemia.

**Table 4:** Summary of indications for agitated saline contrast echocardiography

Summary of Indications for AS-C TTE
**Right to left shunting** resulting in thromboembolic/gas-emboli/arterial hypoxaemia Patent foramen ovale* (intracardiac)
*-resulting in left circulation thromboembolism (cerebral*,* coronary*,* visceral or peripheral infarction)*
*-resulting in decompression illness*
*-resulting in* arterial deoxygenation syndrome (POS**, OSAS, COPD, Exercise desaturation, HAPO)
*-post PFO closure*,* to assess for residual shunts*
Intra-pulmonary shunting (extracardiac)
*- pulmonary arteriovenous malformation*
*- hepatopulmonary syndrome*
Other scenarios
*Neurosurgery in the sitting position- risk of paradoxical air embolism (presence of PFO may be a contra-indication)*
**Persistent left superior vena cava** *** **Complex congenital heart specific scenarios** e.g. baffle leak
**Contrast enhancement of tricuspid regurgitation Doppler velocity signal**
**Catheter position in the correct cavity** e.g. pericardiocentesis

Notes: *Agitated saline contrast TTE is not routinely used in the diagnosis of atrial septal defects where the right heart is dilated. However fenestrated defects of the atrial septum with or without atrial septum aneurysm can be associated with a similar clinical presentation to PFO, see text for details

** *POS* causes are described in Table 3

***Dilated coronary sinus (CS) with normal right heart chambers, contrast seen to fill CS first then right heart following left arm injection

*POS*, platynpnoea orthodeoxia syndrome; *OSAS*, obstructive sleep apnoea syndrome; *COPD*, Chronic obstructive pulmonary disease; *HAPO*, High altitude pulmonary oedema

**Table 5:** Causes of arterial deoxygenation syndrome, where R to L shunting allows deoxygenated blood to enter the left arterial circulation resulting in hypoxaemia

*R* to L shunting	Defect	Mechanism
*Cardiac* *Pulmonary*	Atrial septum abnormality (PFO, ASD, fenestrated septum) Pulmonary AV malformation Vasodilation of pulmonary capillary bed	Typically in setting of raised RAP due to PH Less commonly, in absence of PH, redistribution of blood flow caused by anatomic distortion e.g. proximal aorta dilatation, pericardial disease, (such as loculated pericardial effusion or pericardial constriction), pneumonectomy, kyphosis. Vascular communication between pulmonary artery and vein, typically seen in HHT patients. High flow state through pulmonary capillary bed resulting from vasodilation (thought secondary to nitric oxide levels) resulting in reduced surface area for RBC oxygenation in relation to capillary diameter and increased transit time. Eg liver cirrhosis with hepatopulmonary syndrome,
**Ventilation- perfusion mismatch**		
Lung parenchymal disease*	Redistribution of blood flow within lung	Change in pulmonary blood flow distribution with changes in posture position, resulting in mismatch between ventilated lung and degree of available blood flow to each region (basal versus apical ). Eg emphysema

*Note: conditions related to hypoxaemia must be excluded including pneumonia, pulmonary oedema, adult respiratory distress syndrome and pulmonary embolus

RAP right atrial pressure; PH pulmonary hypertension; HHT Hereditary haemorrhagic Telangiectasia

Similar to the European position document [2], the recent UK Stroke Guidelines (National Clinical guideline for stroke 2023 www.strokeguideline.org) and British Cardiovascular Intervention Society position statement [3] recommend closure of PFO in patients under 60 with no other causative factors for stroke. It must be remembered that a PFO is present in up to 25% of the normal population. Its presence is not automatically indicative of its role in causing stroke. In those under 60 it should only be sought once all other causes of stroke have been excluded. The identification of a PFO and then decision not to close it, due to incorrect patient selection, may lead to patient distress and should be avoided. Further, a PFO should not be sought in those individuals post stroke who are over 60 unless requested following expert structural heart team opinion (or as part of a prospective registry or research trial). It is important to note several contra-indications exist and these are discussed in the next section under *potential risks and limitations*.

Therefore in the setting of stroke, AS-C TTE is indicated when the cause of the stroke is undetermined (cryptogenic) where other causes have been excluded AND where brain imaging is suggestive of a cardioembolic source.

#### Patent foramen Ovale anatomy and imaging planes

**Fig. 1:**
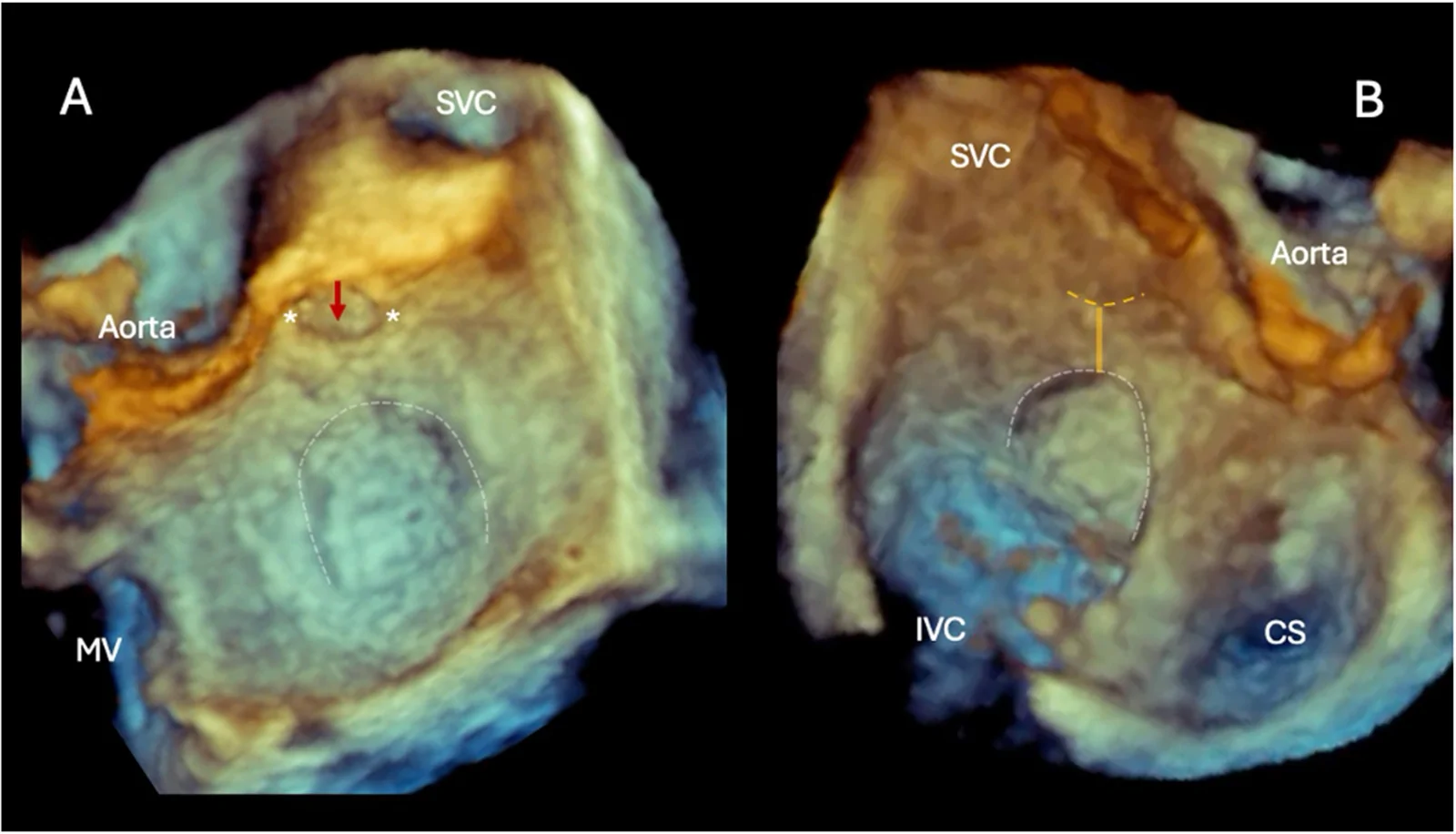
3D TOE anatomy of the atrial septum Image A. enface view of the atrial septum from the left atrium. The septum primum (SP) tissue forms the surface of the left atrial side of the septum, the white stars show attachments of the SP to the septum secundum (SS), while the red arrow points to the PFO opening. Image B enface view of the atrial septum from the right atrium. The SS makes up the right atrial surface of the septum except in the region of white dotted line, which demarcates the edges of the fossa ovalis. The fossa ovalis is covered by the SP. The yellow solid line defines the tunnel of the PFO (where SS and SP tissue overlap) and the yellow dotted line shows where the PFO opens on the left side. *FO fossa ovalis border is marked by a faint dotted white line*. *IVC inferior vena cava*,* SVC superior vena cava*,* CS coronary sinus*,* MV mitral valve*

**Fig. 2:**
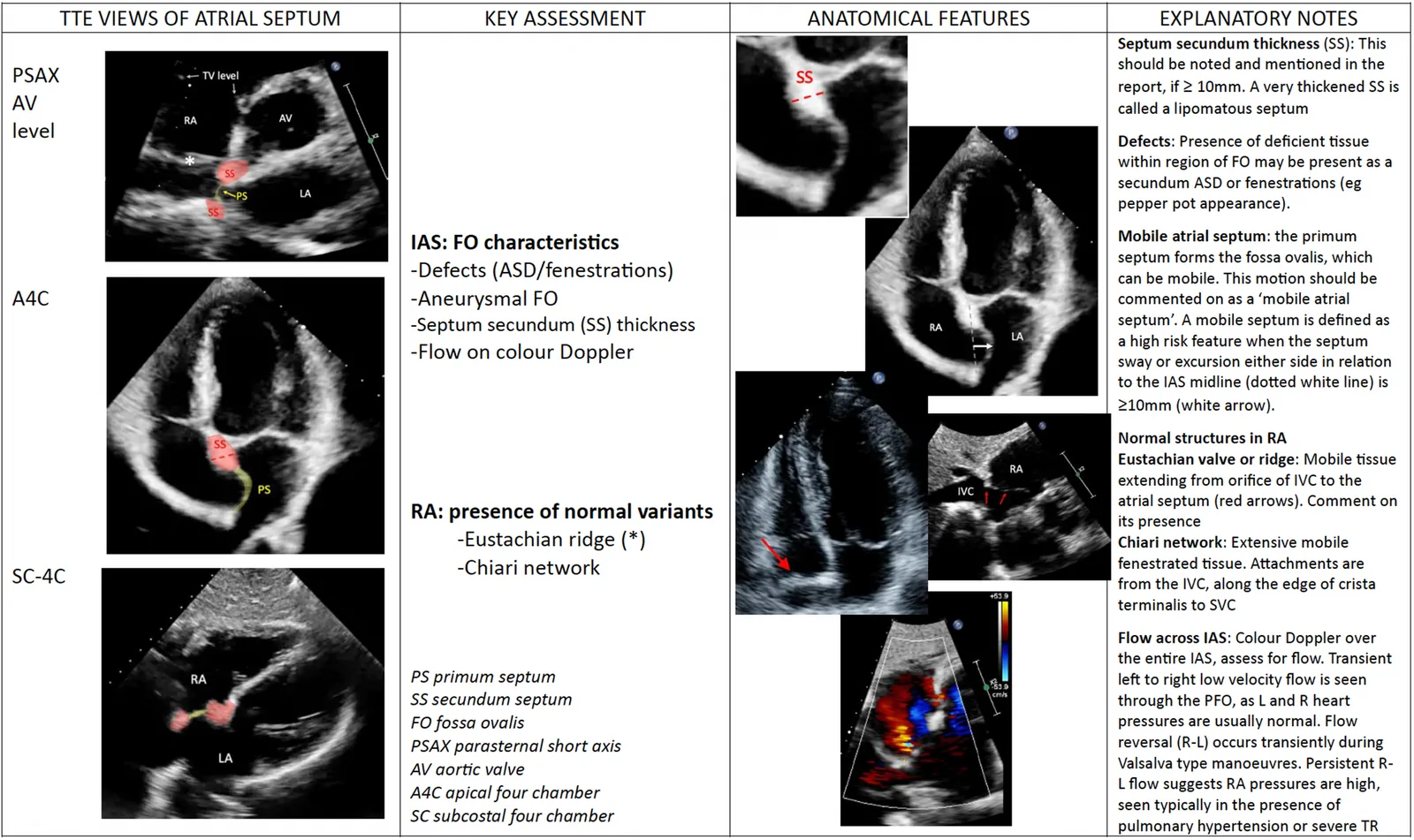
Transthoracic echocardiography protocol. The key transthoracic echo views are depicted along with the necessary parameters which should be described as part of the protocol. *PS primum septum*,* SS secundum septum*,* FO fossa ovalis*, *PSAX parasternal short axis*,* AV aortic valve*, *A4C apical four chamber*, *SC subcostal four chamber*,* IVC inferior vena cava*,* SVC superior vena cava*,* R right*,* L left*,* LA left atrium*,* RA right atrium*,* TR tricuspid regurgitation*,* ASD atrial septum defect*,* PFO patent foramen ovale*

**Fig. 3:**
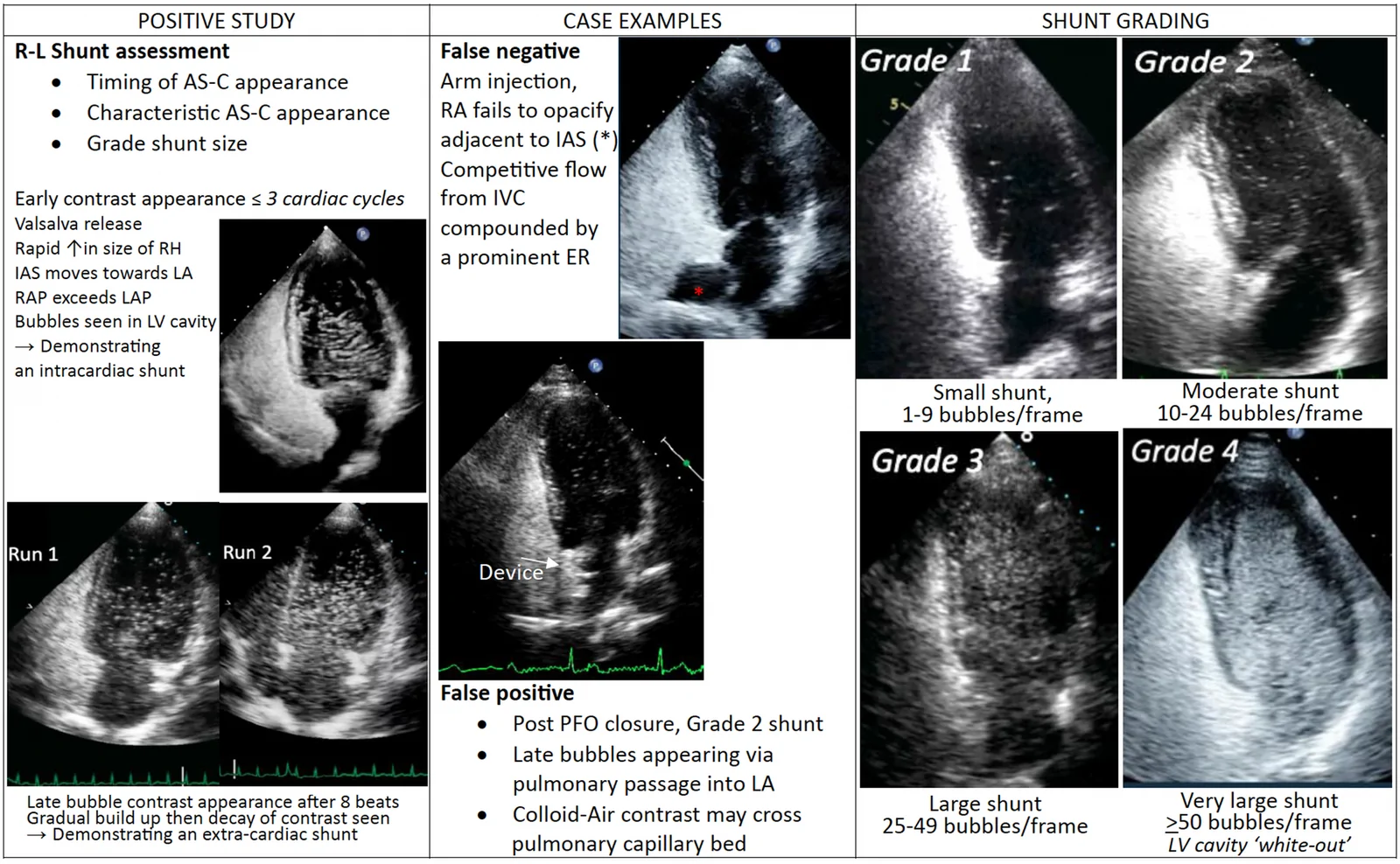
Agitated Saline Contrast Transthoracic echocardiography protocol. Diagnosis of a right to left shunt is described in the left panel. Case examples are depicted in the middle panel and describe commonly seen scenarios associated with false positive and false negative studies. The far right panel shows examples of shunt grades 1–4. *R-L right to left*,* LA left atrium*,* RA right atrium*,* RAP right atrial pressure*,* LAP left atrial pressure*,* RH right heart*,* IAS inter-atrial septum*,* ER Eustachian ridge*,* PFO patent foramen ovale*,* IVC inferior vena cava*

A PFO results from failure of fusion of the septum primum (SP) to the septum secundum (SS). The SS comprises infolded tissue making up the right atrial (RA) side of the atrial septum, but where a portion of the SS is deficient (the infero-posterior region). The SP forms the left atrial side (LA) of the septum, covering the deficiency of the SS. Viewed from the RA the SP tissue is visible where the SS is deficient, forming the fossa ovalis, see Fig. 1. The opening of the PFO tunnel is located somewhere along the superior border of the fossa ovalis. The PFO exit point into the LA is variable but typically is seen directed somewhere between the aorta to the superior vena cava. Precise anatomical description of the PFO tunnel is challenging with TTE imaging. Key characteristics which should be noted on TTE imaging include a hypermobile septum, thickened septum secundum, intermittent flow seen in the region of the fossa ovalis with colour flow Doppler and presence of a prominent Eustachian ridge [4] or valve, see Fig. 2. The three key imaging views are parasternal short axis at the aortic valve level, apical four chamber and subcostal four chamber, here the thicker septum secundum and thinner septum primum (the fossa ovalis) can be identified, Fig. 2. The PFO opening is therefore nearer the aortic valve and superior portion of the FO. While the focus of the article is TTE, important anatomical features seen on transoesophageal echocardiography (TOE) are summarised in Fig. 3. An explanation of the embryology of the atrial septum [5] and TOE protocol [6] along with anatomical characteristics [7] have been described previously.

#### Importance of identifying high-risk PFO anatomical features

Echocardiography features play a pivotal role in the likelihood a PFO is causal rather than incidental in the aetiology of an embolic event. The RoPE (Risk of Paradoxical Embolism) score uses three key clinical characteristics (cardiovascular risk factors, age and pattern of brain imaging) as a method to categorise the likelihood [8]. The score range is 0–10, where a score of 7 or more predicts the likelihood a PFO may be causal in nature. The average RoPE score in patients randomised to PFO closure in the CLOSE trial was 7.4 [9], where a statistically significant benefit above medical therapy alone was demonstrated. A recent Portuguese study reported a score of ≤ 6 to be associated with recurrent stroke post PFO closure, suggesting the PFO was incidental not causal [10]. A RoPE score of 9 predicts an 88% probability of PFO related stroke. It is important to note, however, a higher RoPE score alone, does not in itself predict a high stroke recurrence rate. Large scale studies validating the usefulness of the RoPE score in recurrence of stroke related PFO are lacking [2]. Therefore, it should only be applied within the wider assessment to aid in the identification of patients who might benefit from PFO closure. An analysis of the published randomised controlled PFO closure trials highlighted how anatomical features could refine this prediction [11]. The PASCAL Classification System (PFO-Associated Stroke Causal Likelihood) [11] combines high-risk PFO anatomical features (a large shunt *> 20 bubbles/frame*, and presence of a highly mobile septum, defined as *> 10 mm excursion)* with a RoPE score threshold of 7 to classify the causal role of a PFO as unlikely (< 7 and no high risk anatomical features), possible (< 7 but with high risk anatomical features *or* > 7 and no high risk anatomical features) or probable (> 7 and with high risk anatomical features). This emphasises the importance of characterising anatomical features during echocardiography imaging to assist in risk stratification. It should be noted the term atrial septal aneurysm has now been replaced by mobile septum, since this accurately describes the anatomical variant and avoids unnecessary anxiety to patients.

### Performing the AS-C TTE procedure

Knowledge of contrast preparation, staffing and training, consent, IV access, image acquisition, the procedure protocol along with pitfalls and limitations are necessary. Background information to each step is discussed below. Prior to performing the AS-C injections, the presence or absence of key anatomical features relating to atrial septum anatomy should be assessed and recorded. The TTE standard views, along with the anatomical characteristics are summarised in Fig. 2.

#### Agitated saline contrast preparation

AS-C consists of air microbubbles which through oscillation in the ultrasound beam, produce enhanced signals and backscatter readily seen during echocardiography imaging. Microbubbles of air, generated from agitating saline, opacify the right heart chambers. They typically have a larger diameter than the pulmonary capillary bed. AS-C is short-lived and unable to cross the pulmonary vascular bed (25–35 μm V capillary diameter) where the air microbubbles diffuse into the lungs. Hence the appearance of air microbubbles in the left heart is indicative of the presence of a R-L shunt, which may be intra or extra-cardiac in origin.

The two main types of AS-C range from saline plus air mixture and saline plus air with the addition of patient’s blood. Historical data demonstrated greater diagnostic accuracy when blood was added to the agitated solution [12]. Adding blood produces slightly smaller and more stable (longer lived) microbubbles with a superior echogenicity than just air and saline mixed together [13]. In contrast, the modern echo machines have significantly improved image resolution with tissue harmonic imaging. Therefore, dependant on operator/institution preference and quality of contrast visualised, either of the two types of mixtures can be used. Where blood is used to augment the contrast, consideration should be given to safety of staff to accidental exposure from spillage. A third alternative is the use of a colloid (also referred to as polygelatine solution e.g. gelofusine) solution agitated with air. This solution does not require the addition of blood to create a stable echogenic contrast solution. In some instances it may be the preferred contrast solution, such as in situations where echo windows are poor. However, operators should be aware there are potential limitations and pitfalls to its use (see section below) and it is not recommended for first-line use.

#### Staffing and training for the procedure

Appropriately experienced staff, using the correct equipment both for personal protection and when preparing the contrast is essential (face visor, aprons, gloves). Where possible, performing dedicated lists of AS-C studies assists in developing experience, supports training and quality standards. All staff should be familiar with their local standard operating procedure. Two operators are needed to perform the study. One who scans (sonographer or doctor) and the second who prepares and administers the contrast solution (sonographer, nurse or doctor). The equipment needed to prepare the AS-contrast solution include two luer lock (screw top) 10 ml syringes and a 3 way tap, along with saline solution. The step by step protocol of contrast preparation, contrast injection, image acquisition and shunt grading are described below. For a summary of staff competencies see Table 7.

**Table 6:** Physiology and Nurse Training requirements for performing agitated saline contrast TTE

Training components and competency
An understanding of AS-C microbubble physics
Understanding the indications when and when not to perform/contra-indications AS-C studies
Training in IV access, safety and risks, with potential complications; includes completion of the appropriate Trust competency/certification process
Understanding safety and risks of performing AS-C, with appropriate life support training (ILS)
Standards for correctly performing and reporting AS-C TTE study

In the UK, administration of agitated saline contrast with or without blood is considered a drug. The Human Medicines Regulations 2012 do not authorise nurses, or other registered health care professionals, who are not qualified prescribers to administer prescription only medicines unless an appropriate instruction is in place (BMA policy directorate 2016). Therefore if a doctor is not present during the procedure a patient specific direction (PSD) instruction is required [1]. An example of such a document template, which is necessary for each individual patient is included in the appendix.

#### Consent

Verbal consent should be taken and documented as part of the standard reporting process. This should include an explanation of why the study is necessary, how it will be performed and the need to gain IV access (if not already performed). The safety profile of the procedure is excellent. However, inappropriate application may carry potential risks in specific patient groups, see relevant section below for details. If gelofusin or similar proteinaceous product is used the consent process should highlight that there is a very small risk (approximately 1 in 10,000) of an allergic reaction.

#### Intravenous access

Intravenous access (IV) is gained preferably in a large vein of the arm (antecubital fossa or forearm), although small veins may still be used with good effect. Unless the presence of a left sided superior vena cava is suspected (left arm preference) either arm is acceptable. The patient is positioned on their left side so the right arm is often easier to access during AS-C injection.

#### Image optimisation and acquisition setting

The apical four chamber (A4C) view is usually the preferred imaging window. Ideally, the atrial septum (IAS) is visualised, and slight foreshortening may be necessary. Ensure gain settings are optimised to provide clear differentiation between blood-pool (black) and bubbles (white). High gain settings increase the risk of false positive findings. Tissue harmonic settings should be switched on to improve bubble contrast detection in the left heart (LH). Alternative views where the right heart (RH) is not positioned between the transducer probe and LH may be a reliable alternative when imaging windows are challenging. When the RH overlies the LH, shadowing from the RH contrast will obscure the LH cavities.

If image acquisition is performed prospectively, the image capture should be set to a minimum of 10–15 beat acquisition loop. Acquisition should begin just prior to contrast entering the RH and should capture at least 10 beats following contrast appearance. Retrospective acquisition is best done by freezing the image and scrolling back to 1–2 cardiac cycles prior to contrast appearance in the RH, then capturing the loop. Each stage of the protocol should be labelled before acquisition as ‘resting’, ‘Valsalva’, ‘cough/sniffs’ and ideally the type of contrast used ie saline only, blood/saline, gelofusin. This will avoid any confusion as to which contrast solution and whether manoeuvrers were performed when reviewing the study.

#### Protocol

The main steps of the AS-C TTE procedure are summarised in Table 6, and discussed below.

**Table 7:** Summary of agitated saline contrast TTE protocol and reporting template

Agitated Saline Contrast Transthoracic Echocardiogram Protocol
**Protocol Steps 1–5**
STEP 1: Consent and IV access. Explain to the patient the reasons for the study and the need to gain IV access (if not already done). STEP 2: Gain IV access (appropriately trained staff) STEP 3: Image optimisation and acquisition setting A4C view (alternatives if poor echo window are any view where RH is not positioned between probe and LH, e.g. SC4CV or PSAX LV level). Set acquisition at least 10 cardiac cycles (to include 1–2 beats preceding contrast appearance), or freeze and scroll back to capture injection run STEP 4: Contrast preparation Contrast options: Saline-Air Solution: 7-9 ml saline + 1 ml air Blood-Air-Saline solution: 1 ml blood + 7-8 ml saline + 1 ml air (*Colloid-Air solution: 7-9 ml Gelofusin + 1 ml air) Preparation: Equipment needed, two Luer lock 10 ml syringes and 3 way tap, saline (or gelofusin) solution Fill one syringe with normal saline attach and flush 3-way tap system Syringe 1, fill with 7-8 ml saline (or gelofusin), draw in 1 ml air, then attach to 3 way tap, connect to patient. If Blood-Air-Saline solution then draw back 1 ml blood. Syringe 2, attach the empty syringe to 3 way tap as well, then agitate solution between the two syringes, being careful to be gentle & avoid discomfort to the patient STEP 5: Protocol Perform 10–15 cardiac cycle loop acquisitions at rest then with manoeuvrers *Resting*: optimise echo view (usually A4C), agitate solution, close 3-way tap to allow solution injection to patient and after warning patient of injection, empty syringe *Valsalva manoeuvrer*: Ask patient to freeze breathing (wherever they are in their breath cycle and therefore without taking a deep breath in) and bare down as if straining. Then repeat contrast injection as above. As contrast is injected and is seen to enter the right heart, ask the patient to release their breath-hold but to breath normally and not to take a deep breath. This will help maintain the echo window and allow accurate recording of the pressure changes within the RH and documentation of any bubble contrast passage into the LH. repeat contrast injections 2–3 times or until satisfied adequate quality manoeuvres with RH pressure increases have been performed. *Cough & Sniff manoeuvrer*: Ask patient to take short sharp sniffs and coughs and repeat contrast injections 2–3 times or until satisfied adequate quality manoeuvres with RH pressure increases have been performed. *Note: when Saline-Air +/- Blood contrast solution cannot adequately opacify the RH due to poor contrast solution quality or poor echo windows, an alternative may be to try agitated colloid solution e.g. Gelofusine-Air. However, this should be used with caution since over agitation can result in a very small bubble size which is likely to cause false positives through trans-pulmonary passage. In addition, there is a 1:10,000 risk of anaphylaxis when using any colloid solution and therefore appropriately trained staff should be available if needed during the study. *Positive study* Presence of an intra-cardiac shunt defined as contrast appearance typically in the LH within 3 cardiac cycles after entering the RH, or after release of the Valsalva/cough & sniff. Importantly microbubble contrast appearance has a phasic characteristic when entering the LA via a PFO. Although if a very large shunt if may completely fill the LH and may be difficult to see. Late microbubble contrast appearance** in the LH may be intra-pulmonary shunting (PAVM) or transpulmonary passage (vasodilated pulmonary capillary bed e.g. Hepatopulmonary syndrome, HPS). **Note: Late bubble contrast appearance when due to a PAVM or HPS has a characteristic appearance. Contrast gradually builds in density over several cardiac cycles, then gradually decays away. This build-up and then decay mirrors contrast passage through the RH into the lung vessels and then entering the LA and LV. False positives: -Late microbubble appearance through small bubble size transversing the pulmonary capillary bed. Most often seen with Gelofusin solution. Typically, a puff of bubbles are seen in the LH when recording consecutive contrast injections due to contrast hold up in the lungs. -Mistaking MV subvalve apparatus as bubbles. False negatives: -Poor quality echo windows or contrast solution -Large prominent Eustachian valve/ridge diverting contrast passage away from atrial septum and therefore removing the possibility of contrast to pass through a PFO if prevent *Grading shunt size*, see Table 8 for full details. *Grading the shunt size* is performed using a scale 1–4, taken as any one single still frame.
**Reporting Template**
Reporting follows the standard BSE reporting template. In addition, in the conclusion the following should be summarised: *Baseline TTE IAS characteristics*: Non-mobile *or* mobile, if so is there presence of a high risk feature (≥ 10 mm septum sway) Thick septum secundum (≥ 10 mm) No flow seen *or* Flow across IAS on colour flow Doppler- if so is this intermittent or continuous; is the flow L-R or reversed R-L Presence of Eustachian valve or Chiari network *AS-C TTE* Verbal consent gained IV access (L or R arm, antecubital fossa/foremarm/hand) Agitated saline contrast administered (saline/air/blood) X times, Y time at rest, Z times with Valsalva (and sniff/coughs) Positive study seen at rest (or only after manoeuvres) with early or late contrast appearance (describe within how many cardiac cycles contrast appearance seen) or negative study (rest and after manoeuvres) Shunt grade at rest Shunt grade with Valsalva and/or sniff/coughs Summary: Positive (or negative) AS-C study, with early *or* late bubble contrast appearance associated with a grade [1 to 4] shunt.

##### Resting study

The echo image is optimised prior to injection. Warn the patient you will inject the agitated saline contrast rapidly into the arm. Ensure the three-way tap is closed to the empty syringe and immediately inject contrast. The aim is to demonstrate contrast entering the RH, completely opacifying the right atrium (RA) with dense contrast along the entire length of the interatrial septum. Acquire the image loop as described above. If the study is negative (no ‘microbubbles’ seen to appear in the LH), or positive without the shunt size being graded as large, then proceed to performing the AS-C injection again with provocative manoeuvres.

##### Provocative manoeuvres

Normal pressures in the left chambers exceed the right heart chambers. Therefore, the reliance on the appearance of contrast in the LH to diagnose the presence of an intra-cardiac shunt is dependent on the pressure gradient transiently reversing to allow contrast to pass through the opening into the LH. A PFO has a flap-valve anatomy, where typically it is closed unless the RA pressures exceed left atrium (LA) pressures forcing the flap-valve open allowing flow from the RA to enter the LA. The purpose of a Valsalva manoeuvre is to temporarily reverse pressures between right and left atrium to unmask the presence of an intra-cardiac shunt. Hence, if the resting contrast study is negative or a shunt is seen but is not categorised as large (Grade 3–4, see grading system below) then it is necessary to repeat the process now with Valsalva. Ask the patient to stop their breathing (wherever they are in their breath cycle and therefore without taking a deep breath in) and bear down as if straining. Then repeat contrast injection as above. As contrast is injected and is seen to enter the RH, ask the patient to release their breath-hold but to breathe normally and not to take a deep breath. This will help maintain the echo window and allow accurate recording of the pressure changes within the RH and documentation of any AS-C passage into the LH. To ensure this is done correctly it is often useful to practise the manoeuvre with the patient prior to contrast injection. There are several variations on how to best perform a Valsalva. Some patients may struggle to follow instructions. Therefore an alternative method may be to instruct the patient to blow into the connecting end of an empty syringe with the plunger fully depressed into the barrel. Evidence the RA pressure has exceeded the LA pressure is supported by the observation that the RH has reduced in size and the IAS bows towards the LA as the Valsalva is released. Repeat contrast injections 2–3 times or until satisfied manoeuvres of adequate quality have been performed (and RH pressure increase has been observed). Published data suggests the presence of a shunt can be missed despite performing the Valsalva [14]. Therefore, it is advised to add cough and sniff manoeuvrers if the test is negative, or a shunt is seen but not classed as large. Ask the patient to take short sharp sniffs and coughs and repeat contrast injections 2–3 times or until satisfied adequate manoeuvres with RH pressure increases have been performed [6]. In a ventilated patient ask the anaesthetist to perform a valsalva (typically by closing the adjustable pressure limiting valve and increasing airway pressure either by increasing inspiratory pressure or positive end-expiratory pressure) maintain the echo image, then inject the AS-C immediately followed by Valsalva release.

#### A positive study

##### Early appearance of microbubbles in LH

The presence of an intra-cardiac shunt is defined as contrast appearance in the LH typically seen within 3 cardiac cycles after entering the RH, either at rest or following release of the Valsalva/cough&sniff. However, the timing of contrast appearance in the LH is dependent on flow reversal and hence the characteristic of AS-C appearance is important to define. An intra-cardiac shunt has a phasic characteristic when entering the LA via a PFO. Although, in the case of a very large shunt, contrast may completely fill the LH rapidly making it difficult to decipher the nature of the flow.

##### Late appearance of microbubbles in LH

Presence of AS-C appearance in the LH when seen after 3 cardiac cycles from the time contrast enters the RH is defined as late appearance of microbubbles. It is indicative of intra-pulmonary shunting (pulmonary arteriovenous malformation, PAVM) or transpulmonary passage (vasodilated pulmonary capillary bed e.g. Hepatopulmonary syndrome, HPS). Late AS-C appearance when due to a PAVM or HPS has a characteristic appearance. Contrast gradually builds in density over several cardiac cycles, then gradually reduces. This build-up and then decay mirrors contrast passage through the RH into the lung vessels and then entering the LA and LV and is distinct from the phasic flow typically observed through a PFO.

#### Potential risks, limitations and contraindications

AS-C echocardiography is a safe technique [15]. However, there are limited case reports and case series describing potential complications relating to AS-C studies. Romero et al. [16] reported 5 cases of cerebral ischaemic events during or very soon after the AS-C study. The event rate appears to be extremely small and is typically described in the literature in specific patient cohorts, i.e. older patients (in Romero et al. [16] 2 of the 5 cases included an 80 and 90 year old patient), those with significant pulmonary hypertension [17] and those with atrial septal defects [18]. AS-C studies should not be performed in high-risk groups and emphasises the importance of appropriate patient selection. With improved contemporary understanding of disease states and superior quality imaging techniques, patients with pulmonary hypertension, atrial septal defects (or complex congenital disease) and the elderly should NOT undergo AS-C TTE, unless discussed with the appropriate cardiologist expert in performing AS-C studies. For the current indications, AS-C TTE is considered safe and does not pose a risk to the patient. Anecdotal references to migraine experienced soon after (< 24 h) have been suggested. However, there is no published data to support this phenomenon being related to the use of AS-contrast. As with any venous injection carefully ensuring there are no visible air bubbles (additional to a microbubble solution) within the syringe prior to injection is paramount. Limited published data exits for the application of AS-C TTE during pregnancy. However a recent retrospective review of 100 pregnant women found no adverse events (air embolism or foetal distress) relating to the use of AS-C TTE (indicated for stroke in the majority) [19]. Therefore, AS-C TTE may be safely performed during pregnancy if clinically indicated [20].

Gelofusine or similar colloid preparation contains gelatine. When this is agitated with air (and thereby avoiding the use of blood) it produces a highly echogenic solution. It may be the preferred contrast for this reason when blood cannot be drawn into the saline-air mixture or despite adequate contrast quality it is poorly visualised during limited TTE windows. However, it is not recommended for standard use as there is a higher false positive incidence as the colloid microbubbles generated can typically cross the pulmonary capillary bed. Since it contains protein material, rarely it can result in hypersensitivity reactions. Therefore, staff administering the solution should be appropriately trained.

**Fig. 4:**
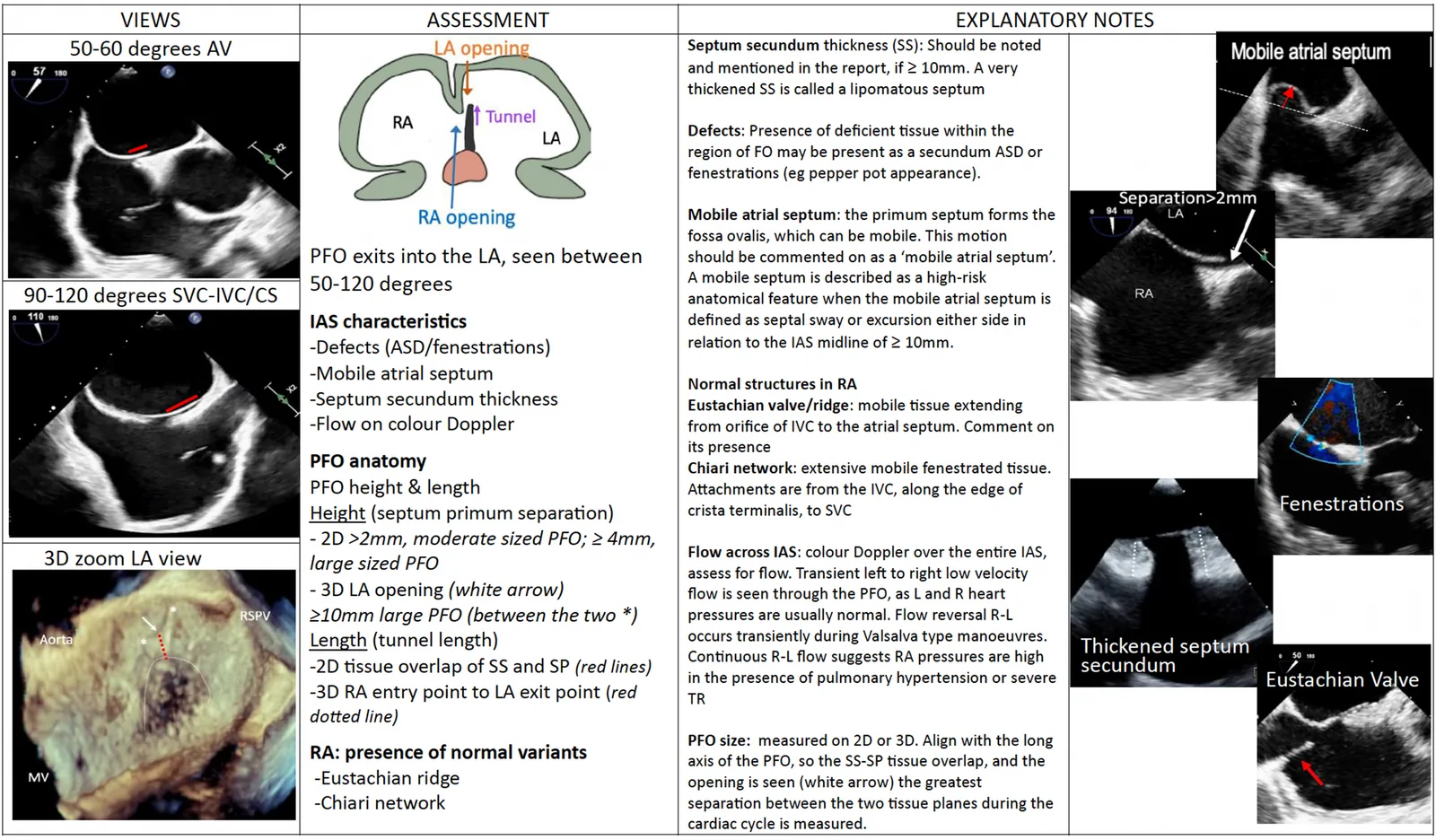
Transoesophageal echocardiography protocol. The key transoesophageal echo views are depicted along with the necessary parameters which should be described as part of the protocol. *AV aortic valve*,* CS coronary sinus*,* MV mitral valve*,* RSPV right superior pulmonary vein*,* LA left atrium*,* RA right atrium*,* ASD atrial septal defect*,* IVC inferior vena cava*,* SVC superior vena cava*,* PFO patent foramen ovale*,* PS primum septum*,* SS secundum septum*,* IAS inter-atrial septum*,* TR tricuspid regurgitation*,* 2D two-dimensional*,* 3D three-dimensional*,* R right*,* L left*

False positives include:

- Late bubble appearance may result from a smaller bubble size of the AS-C transversing the pulmonary capillary bed. The late appearance of a puff of bubbles may be seen in the LH when recording consecutive contrast injections due to contrast hold up in the lungs. When using gelofusine care must be taken not to over agitate the solution with air. There is a tendency to produce increasingly smaller bubbles which readily transverse the lungs and can give false positive studies. If in doubt revert to saline-air (with or without blood) solution to ensure the results are reproducible. Where doubt remains then a TOE will differentiate flow from the pulmonary veins versus flow through the IAS, Fig. 4.
- Mistaking mitral subvalve apparatus as bubbles. Therefore, careful review of the baseline TTE and the corresponding echo view is essential to avoid over interpretation.

False negatives include:

- Poor quality echo windows or poor contrast solution resulting in contrast being poorly visualised and a shunt is missed.
- Large prominent Eustachian valve/ridge diverting contrast passage away from the atrial septum. Therefore, preventing AS-C passing through a PFO if present. In this situation cannulation of a leg vein and AS-contrast injection via the IVC (where blood flow is directed preferentially towards the IAS) may overcome this challenge. However, this can be considered invasive and may cause more discomfort to the patient. Alternatively, proceeding to a transoesophageal echocardiogram (TOE) may be more appropriate.
- Despite performing provocative manoeuvrers the IAS fails to shift towards the LA, and persistently bows towards RA. There will be uncertainty as to whether the atrial pressures adequately reversed to allow AS-C passage through a potential PFO. If attempts at provocative manoeuvres cannot confirm RA pressure exceeded the LA pressure, then a TOE may be indicated.

#### Grading shunt size

The amount of AS-contrast seen in the LH is arbitrarily quantified by the largest number of bubbles seen in the LH in a still frame. The major randomised controlled trials used some variation in the thresholds. However, a metanalysis of these trials demonstrated a statistically significant higher risk of stroke in those with a large shunt [21]. The range for a small shunt was 5–10 and large 20–30 bubbles [22]. Therefore reflecting on the literature [9, 23–25] and considering the clinical implications of shunt grading, a practical approach is proposed [6], see Table 8; Fig. 4. The shunt size is defined in terms of grades 1–4, with both a subjective and objective description for each grade. Grading the shunt size is performed using any one single still frame.

**Table 8:** Shunt grading of agitated saline contrast appearance in the left heart [6]

Grading of right-to-left interatrial shunts	
Grade 1	Subjective: A few scattered bubbles seen individually within the left atrium or ventricle Objective: No more than 5 bubbles in one still frame
Grade 2	Subjective: Clusters of bubbles seen in left atrium or ventricle Objective: 5–25 bubbles in one still frame
Grade 3	Subjective: Clouds of bubbles seen in left atrium or ventricle, without complete opacification Objective: >25 bubbles in one still frame but still countable
Grade 4	Subjective: Complete opacification of left heart chamber (left atrium or ventricle) Objective: Bubble contrast confluent, typically > 50 bubbles one still frame

#### Reporting template

When a standard TTE is performed for a possible cardioembolic source, if a cardiac lesion is found this should be reported as per standard guidelines. If the heart is structurally normal, it is helpful to include in the summary specific mention of the atrial septum anatomy. Particular points would include, presence or absence of a mobile atrial septum, any flow seen across the fossa ovalis using colour flow Doppler, the direction of flow if seen (e.g. R to L) and whether it is continuous or intermittent.

When reporting a AS-C TTE study, the summary should clearly state whether the study was positive or negative. Following this statement, a further breakdown of whether there was early or late appearance of AS-contrast within the LH, the grade of the shunt at rest and then following provocative manoeuvres. The summary should conclude with a diagnosis of intra-cardiac or extra-cardiac shunt or no shunt seen, see Table 6 reporting section for further details.

### Other imaging modalities in the diagnosis of a right to left shunt

An AS-C study can be performed to assess the shunt by TOE. Various metanalyses have compared the use of AS-C during TOE, TTE and transcranial Doppler (TCD). The data is inconsistent and of limited quality. A metanalysis comparing TCD with TOE found TCD had a sensitivity and specificity of 94% and 92% respectively [2]. The same group performed a similar metanalysis comparing the diagnostic yield of TTE compared to TOE and found a sensitivity of 88% and specificity of 82% [2]. Other metanalyses have found TCD when compared to TTE to be marginally superior in shunt detection, particularly small shunts [26, 27]. Each modality has its advantages and disadvantages. TCD assessment is more cost effective and is quantitative (large shunt = 10 Hits or more) [28] but can be limited in up to 20% due to bone thickness and the location of the shunt cannot be deciphered. TTE provides assessment of the cardiac structures, is able to define the level of the shunt, however, is less sensitive to small shunts and may be limited by poor echo windows. TOE is not necessarily superior to the other two modalities in AS-contrast shunt detection, although this finding is not surprising when considering the limitations of performing provocative manoeuvres while a TOE probe is inserted and the patient under some degree of sedation. An abdominal thrust can be employed but this can be unreliable in reversing RA-LA pressures. The role of AS-C during TOE therefore can have limited value, although the superiority of TOE as the definitive diagnostic modality supersedes the need to demonstrate a shunt using AS-contrast. TOE provides high quality image resolution allowing direct visualisation of the IAS and its components, with detailed views of the PFO [6]. Colour Doppler clearly defines flow within the PFO, while 3D echo imaging offers unrivalled anatomical spatial understanding and defect sizing [29]. Key IAS anatomical parameters should be reported during a TOE study and are summarised in Fig. 3.

The first-line modality of choice is TTE. Where TCD is available and appropriate expertise exists (see D’Andrea et al. [30] and Miceli G et al. [31], for further information), this is an alternative for shunt diagnosis. Where doubt remains, a TOE may be necessary to assess IAS anatomy, as well as ruling out other cardiac sources of embolism. TOE is indicated to assess suitability and determine device choice prior to performing PFO closure. AS-C TOE is useful when there is doubt regarding the presence of a PFO and demonstrating an extra-cardiac shunt (contrast seen to enter the LA via a pulmonary vein) to help explain the AS-C TTE findings.

## Conclusion

Agitated saline contrast TTE is a safe procedure primarily used to diagnose the presence of a right to left shunt. The commonest related clinical syndrome is ischaemic stroke in the younger patient and may be associated with the presence of a patent foramen ovale. Scoring systems help aid risk stratification and specific anatomical PFO characteristics may increase the likelihood that the PFO may play a causal role. A number of limitations and pitfalls exist and therefore a systematic approach to performing and reporting the AS-C TTE is essential to mitigate the risks of misinterpretation, thereby ensuring delivery of both a high-quality echocardiographic study and AS-C service.

## Electronic supplementary material

Below is the link to the electronic supplementary material.

Supplementary material 1

## Data Availability

Not applicable.
