# Correction: Agitated Saline Contrast Echocardiography in the diagnosis of right to left shunts: Guidance and recommendations from the British Society of Echocardiography

**DOI:** 10.1186/s44156-026-00128-0

**Published:** 2026-09-25

**Authors:** Bushra Rana, Shaun Robinson, Liam Ring, Dave Oxborough, Mark Belham, Jim Newton, Alfa Ali, Nilesh Sutaria, Nina Bual, Martin Swaans, Nina Wunderlich, Victoria Delgado, David Northridge, Dave Hildick-Smith, Neil Ruparelia, Mark Turner, Michael Mullen, Chris Baker, Brian Clapp, Mark Spence, Horst Sievert, Iqbal Malik, Roxy Senior, Anjana Siva

**Affiliations:** 1 Guys and St Thomas’ Hospital NHS Foundation Trust, London, UK; 2 Imperial College Healthcare NHS Trust, London, UK; 3 West Suffolk Hospital NHS Foundation Trust, Bury St Edmunds, Suffolk, UK; 4 Research Institute for Sports and Exercise Science, Liverpool John Moores University, Liverpool, Merseyside, UK; 5 Addenbrookes Hospital, Cambridge University Hospitals NHS Foundation Trust, Cambridge, United Kingdom; 6 The John Radcliffe Hospital, Headley Way, Oxford, UK; 7 Department of Cardiology, St. Antonius Hospital, Nieuwegein, the Netherlands; 8 Department of Cardiology/Angiology, Asklepios Klinik Langen, Langen, Germany; 9 Hospital University Germans Trias i Pujol, Badalona, Spain; 10 Institute for Health Science Research Germans Trias i Pujol (IGTP), Badalona, Spain; 11 Edinburgh Royal Infirmary, Edinburgh, Scotland, UK; 12 Royal Sussex County Hospital, Brighton, UK; 13 University Hospital Bristol NHS Foundation Trust, Bristol, UK; 14 St Bartholomew’s Hospital, London, UK; 15 Mater Private Hospital, RCSI University of Medicine & Health Sciences, Dublin, Ireland; 16 CardioVascular Center Frankfurt, Frankfurt, Germany; 17 Royal Brompton Hospital, London, UK; 18 Queen Alexander Hospital, Portsmouth, UK

**Correction to: Echo Res Pract (2026) 13:16**

**https://doi.org/10.1186/s44156-026-00117-3**

Following publication of the original article [1], the authors identified errors in Fig. 1. Both the incorrect and correct information is given hereafter. It incorrect read in the figure caption ‘3E TOE anatomy of the atrial septum’ instead of ‘3D TOE anatomy of the atrial septum’ and a paste icon was present.

The incorrect Figure 1:

**Fig. 1:**
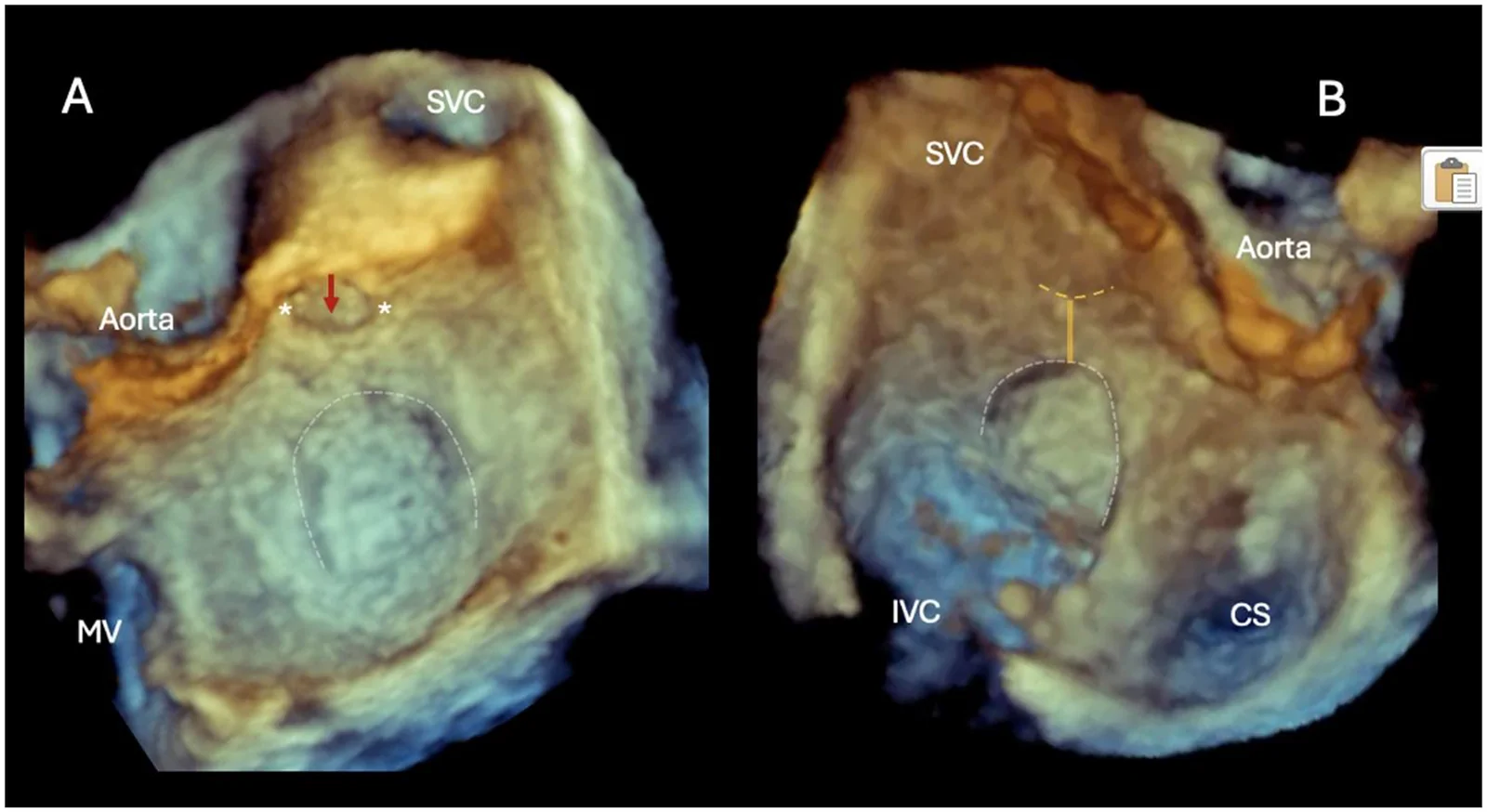
3E TOE anatomy of the atrial septum Image A. enface view of the atrial septum from the left atrium. The septum primum (SP) tissue forms the surface of the left atrial side of the septum, the white stars show attachments of the SP to the septum secundum (SS), while the red arrow points to the PFO opening. Image B enface view of the atrial septum from the right atrium. The SS makes up the right atrial surface of the septum except in the region of white dotted line, which demarcates the edges of the fossa ovalis. The fossa ovalis is covered by the SP. The yellow solid line defines the tunnel of the PFO (where SS and SP tissue overlap) and the yellow dotted line shows where the PFO opens on the left side. *FO fossa ovalis border is marked by a faint dotted white line. IVC inferior vena cava, SVC superior vena cava, CS coronary sinus, MV mitral valve*

The correct Figure 1:

**Fig. 1:**
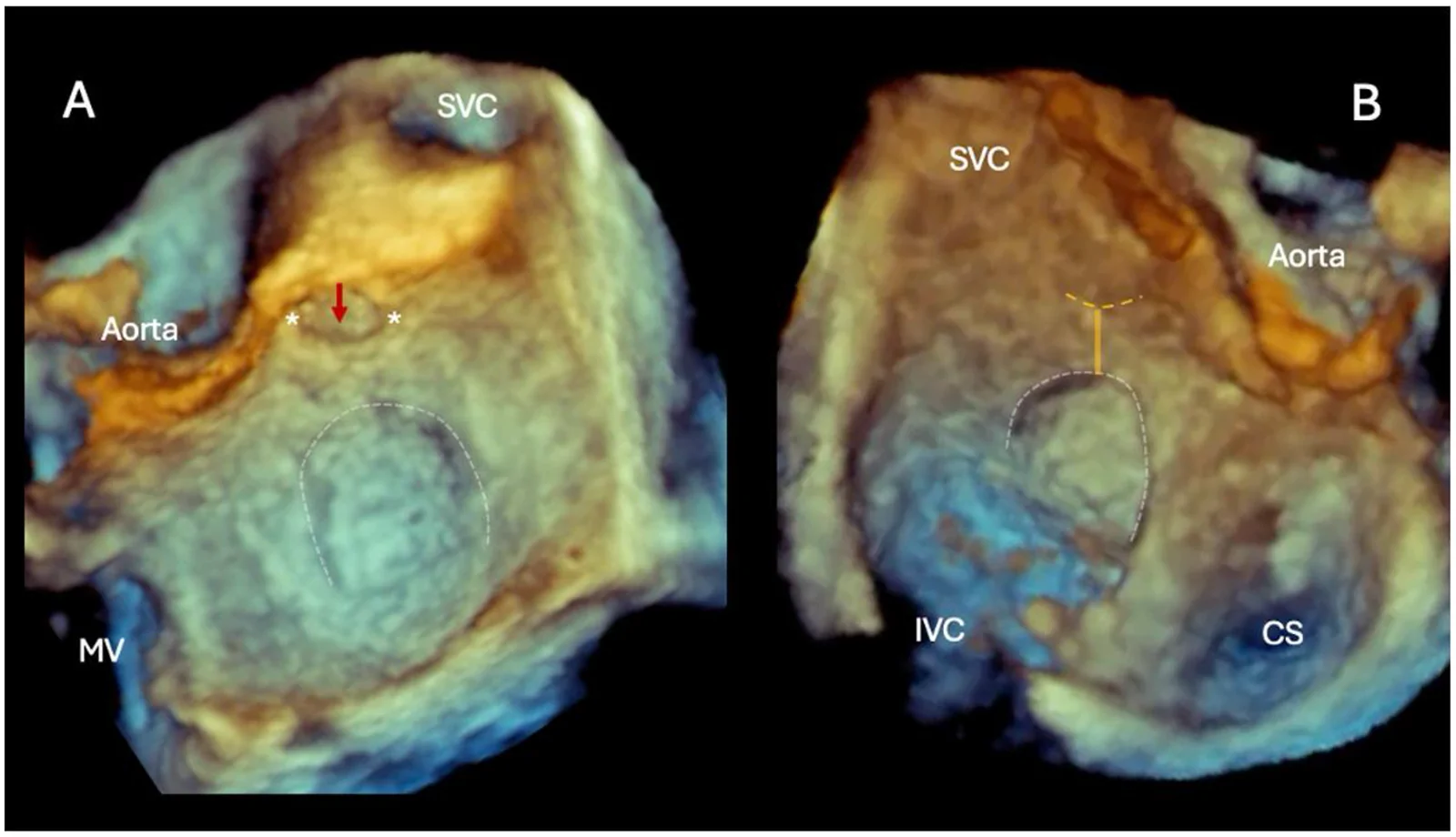
3D TOE anatomy of the atrial septum Image A. enface view of the atrial septum from the left atrium. The septum primum (SP) tissue forms the surface of the left atrial side of the septum, the white stars show attachments of the SP to the septum secundum (SS), while the red arrow points to the PFO opening. Image B enface view of the atrial septum from the right atrium. The SS makes up the right atrial surface of the septum except in the region of white dotted line, which demarcates the edges of the fossa ovalis. The fossa ovalis is covered by the SP. The yellow solid line defines the tunnel of the PFO (where SS and SP tissue overlap) and the yellow dotted line shows where the PFO opens on the left side. *FO fossa ovalis border is marked by a faint dotted white line. IVC inferior vena cava, SVC superior vena cava, CS coronary sinus, MV mitral valve*

Figure 1 has been updated in this correction article and the original article [1] has been corrected.
