# Supplementary material for: Agitated Saline Contrast Echocardiography in the diagnosis of right to left shunts: Guidance and recommendations from the British Society of Echocardiography
Source: Echo Res Pract. 2026 Jun 1;13:16. doi: 10.1186/s44156-026-00117-3 (PMC13224608; doi:10.1186/s44156-026-00117-3)
Supplement: Supplementary file 1 — Supplementary material 1 [file 44156_2026_117_MOESM1_ESM.doc]

PATIENT SPECIFIC DIRECTION – PSD.

| NOTES ON PSD’s.  A Patient Specific Direction is a legal mechanism by which Medicines and Medical Devices (*[Enter name] Hospital NHS Trust*) can be supplied and/or administered to a specifically identified patient by a specific range of health care professionals, after being assessed by a suitable professional.  *[Enter name] Hospital NHS Trust* recognises the need for good governance and control regarding the administration of all Medical devices. Therefore it must be agreed and signed by the patient’s consultant and the Nurse Specialist prior to use for the specific patient in question. |
| --- |

| Clinical Service Centre - CSC. |  |
| --- | --- |

| Drug: Agitated saline, blood and air bubble contrast (AS-C) | *Area / Discipline*  Cardiac Investigations Unit | *User Code* |
| --- | --- | --- |
|

| Patient Name & ID (Sticker).  Surname : ………………………………….  Forename : ………………………………….  Hosp. Number : ………………………………….  Date of Birth : …………………………………. | Consultant.  Signature : |
| --- | --- |
|  | Cardiac Physiologist  Name:  Signature : ……………………………… Date : …………….. |
| Start Date: | Finish Date: |

| CLINICAL CONDITION |
| --- |

| Clinical Indication/Situation | Agitated saline echocardiography is a diagnostic test used to identify selected patients with a right to left communication e.g. patent foramen ovale (PFO), atrial septal defect or intrapulmonary AV shunt these bubbles pass into the left atrium and are seen during the ECHO scan.  Sodium chloride 0.9%, air and patients own blood are mixed and agitated to generate tiny bubbles (10-100µm). The resultant unlicensed product is injected intravenously and the bubbles can be seen with echocardiography entering the right atrium only where there is communication between the right and left circulation. |
| --- | --- |
| **Inclusion criteria** | All patients referred for AS-C echocardiography will require approval of a Consultant Cardiologist and may include:  *Common indications*   - Selected patients after stroke/TIA - Selected patients with hypoxia - Selected patients with possible paradoxical embolus   *Less common indications:*  Rare consequences of PFO that may result in referral for AS-C echocardiography   - Decompression illness seen in divers - Arterial deoxygenation syndrome where significant quantities of deoxygenated blood passes into the left side of the circulation (platynpnoea orthodeoxia syndrome, obstructive sleep apnoea, COPD, High altitude pulmonary oedema, Exercise desaturation),     Post PFO closure, to assess for residual shunts  Intra-pulmonary shunting (extracardiac)  - pulmonary arteriovenous malformation  - hepatopulmonary syndrome  Neurosurgery in the sitting position- risk of paradoxical air embolism (presence of PFO may be a contra-indication) |
| **Exclusion criteria** | Patients with severe pulmonary arterial hypertension  Patients not formally accepted by consultant cardiologist through procedure triaging process |
| **Cautions/Need for further advice**  **&**  **Risk Control Measures** | Caution – very elderly patients – over 80  (there have been case reports of stroke following bubble contrast in these groups but the risk is still very low). Discussion with Cardiologist required to understand if appropriate indicatation |
| **Action if patient declines or is excluded** | There is no alternative test – discuss with referring consultant |

| MEDICINE DETAILS |
| --- |

| **Pharmaceutical details** | **Composition:** 7-8ml of sodium chloride 0.9% mixed with 1ml air and 1 ml patients own blood drawn from cannula (ideally 20 gauge or larger in antecubital fossa).  This mixture is immediately agitated between 2 luer lock syringes attached to the above cannula to produce a frothy mixture containing bubbles. The luer lock syringes provide a closed system to minimise spillage/spray of blood    **Classification:** Sodium chloride 0.9% POM |
| --- | --- |
| **Route(s)/Method(s) of administration** | Intravenous administration via cannula immediately after preparation of bubble mixture  Gloves and visors worn by team |
| **Dosage** | Approximately 9ml per injection  The injection is repeated 3-5 times with simultaneous echocardiography of the heart performed to see if any bubbles pass into the left atrium from the right atrium after each injection  Provocative manoeuvres eg Valsalva and sniff /cough are performed to raise right atrial pressure and increase sensitivity of the test |
| **Frequency** | A single episode of the above protocol |
| **Duration of treatment** | A single episode of the above protocol |
| **OR Maximum or minimum treatment period** |  |
| **Quantity to be supplied/administered** | Approximately 9-10ml per injection 3-5 times |
| Side effects | No published side effects but anecdotal reports of migraine within 24 hours |
| **Advice to patient/carer** | No effects should be experienced during the test  No precautions needed after the test |
| **Follow up** | No specific follow up needed for AS-C contrast injection  Clinical follow up will be arranged according to the results of the test |

| **STAFF CHARACTERISTICS** |
| --- |

| Qualifications | - RCCP or HCPC Registered Physiologist with a minimum of 2 years experience at Band 6 or higher. - BSE accredited echocardiographer   RCN registered Staff nurse band 5 or above (specific qualifications)  . |
| --- | --- |
| Specialist competencies or qualifications | Training in intravenous access and administration of intravenous drugs  Training in AS-C preparation and injection with sign off after a period of supervised practice  Training in instructing patient on performing Valsalva/sniff manoeuvres |
| Continuing training & education | Audit of cases and complications  Audit of results and quality of echo images obtained |

| **REFERRAL ARRANGEMENTS AND AUDIT TRAIL** |
| --- |

| **Referral arrangements** | As instructed by local PSD steering group |
| --- | --- |
| **Records/audit trail** | 1. Qualifications of person administering or supplying this medical device and assessment record 2. Condition treated / diagnosis 3. If patient excluded, why? 4. If patient excluded/did not wish to be included, what further action taken? 5. Medical Device supplied / administered 6. Doses and number of doses 7. Advice given to patient/relative/carer 8. Further referral arrangements made |

| **REFERENCES** |
| --- |

| **References/Resources and comments** |  |
| --- | --- |

| AUTHOR DETAILSName: **Job Title:**  **Department:**  **Acknowledgements (Co-authors, other establishments)** |
| --- |

| REVIEWED BY SENIOR PHARMACIST This is only required when the device has replaced an item  which was previously listed as a licensed medication. | Signed: ( ) |
| --- | --- |

| AUTHORISATION |
| --- |

This Patient Specific Direction must be authorised and signed by all health care professionals involved in its use The NHS Trust should hold the original signed copy. The PSD must be easily accessible in the clinical setting

| Lead Doctor | **Name:**  **Position:**    **Signature:**  **Date:** |
| --- | --- |
| Lead Nurse or Senior Nurse / Allied Health Professional | Name: **Position:**  **Signature:**  **Date:** |
| Lead Pharmacist | Name: **Position:**    **Signature:**  **Date:** |
| **Clinical Governance Lead** | Name: **Position:**    **Signature:**  **Date:** |
| **PgD Committee Chair** | Name: **Position:**  **Signature: Date:** |
| **Formulary and Medicines Chair** | Name: **Position:**  **Signature: Date:** |

| USER AUTHORISATION |
| --- |

| MEDICAL DEVICE: |
| --- |

**PSDs DO NOT REMOVE INHERENT PROFESSIONAL OBLIGATIONS OR ACCOUNTABILITY.**

It is the responsibility of each professional to practice only within the bounds of their own competence and in accordance with their own Code of Professional Conduct.

**Note to Authorising Managers:** authorised staff should be provided with an individual copy of the clinical content of the PSD and a photocopy of the document showing their authorisation.

I have read and understood the Medical Device Direction and agree to supply/administer this medical device only in accordance with this PSD.

| **Name of Professional** | **Initials** | **Signature** | **Authorising Manager** | **Date** |
| --- | --- | --- | --- | --- |
|  |  |  |  |  |
|  |  |  |  |  |
|  |  |  |  |  |
|  |  |  |  |  |
|  |  |  |  |  |
|  |  |  |  |  |
|  |  |  |  |  |
|  |  |  |  |  |
|  |  |  |  |  |
|  |  |  |  |  |
|  |  |  |  |  |
|  |  |  |  |  |
|  |  |  |  |  |
|  |  |  |  |  |
|  |  |  |  |  |
|  |  |  |  |  |
|  |  |  |  |  |
|  |  |  |  |  |
